# Effects of local extrinsic mortality rate, crime and sex ratio on preventable death in Northern Ireland

**DOI:** 10.1093/emph/eov020

**Published:** 2015-09-03

**Authors:** Caroline Uggla, Ruth Mace

**Affiliations:** Department of Anthropology, University College London, London WC1H 0BW, UK

**Keywords:** preventable death, life history theory, extrinsic mortality rate, crime, adult sex ratio, health behaviour, census

## Abstract

**Background and objectives**: Individual investment in health varies greatly within populations and results in significant differences in the risk of preventable death. Life history theory predicts that individuals should alter their investment in health (somatic maintenance) in response to ecological cues that shift the perceived fitness payoffs to such investments. However, previous research has failed to isolate the effects of different ecological factors on preventable death, and has often relied on macro-level data without individual controls. Here, we test some key predictions concerning the local ecology—that higher extrinsic mortality rate (EMR), crime rate and mate-scarcity (male/female-biased sex ratio) at the ward-level—will be associated with a higher risk of preventable death.

**Methodology**: We use census-based data from Northern Ireland (*n* = 927 150) on preventable death during an 8.7-year period from the 2001 Census and run Cox regressions for (i) accident/suicide or alcohol-related death and (ii) deaths from preventable diseases, for men and women separately, controlling for a wide range of individual variables.

**Results**: We find evidence of ward-level EMR and crime rate being positively associated with preventable death among men, particularly men with low socioeconomic position. There was a tentative relationship between male-biased sex ratio and preventable death among women, but not among men.

**Conclusion and implications**: Both behaviours that might lead to ‘risky’ death and health neglect might be adaptive responses to local ecologies. Efforts to reduce crime might be as effective as those to reduce extrinsic mortality, and both could have positive effects on various health behaviours.

## Abstract (precis)

Variation in preventable death can be understood from an evolutionary life history perspective. However, previous studies have failed to isolate ecological effects on preventable death. We use population-wide Census data from Northern Ireland and find that extrinsic mortality rate and crime, but not sex ratio, impacts on male preventable death.

## INTRODUCTION

Individuals vary greatly in the effort they expend on achieving and maintaining good health. Understanding the root causes of this variation holds great potential to improve population health. It is well-established that a large proportion of health and mortality variation is associated with socioeconomic factors [1, 2]. Individuals with lower socioeconomic position (SEP) have lower life expectancy, lower healthy life expectancy [3, 4], higher incidence of various potentially fatal diseases such as heart disease [5], diabetes [6], lung cancer [7], and higher risk of alcohol-related death [8] and death from injuries [9, 10]. The role of individual behaviour in generating these patterns is indisputable: smoking, drinking and diet-related diseases account for the majority of premature deaths in Western countries and are highest among low SEP individuals [11, 12]. Individuals with higher SEP are healthier for a number of reasons, but in particular seem better positioned to give up behaviours with negative health repercussions and comply with treatment, demonstrating, for example, higher smoking cessation success [13] and better survival following cancer diagnosis than less wealthy peers [14]. These health differentials exist even in the presence of universal access to health care and widespread knowledge through public health campaigns about adverse effects of poor diet, smoking and excessive alcohol consumption [15].

A large body of literature examines the association between an individual’s local environment and her risk of death. These ‘area effects’ are of great interest to policy makers as evidence of the influence of the local environment could help shape public health interventions. To date however, the results from studies analyzing area effects are mixed [5]. A recent review of the literature finds negative associations between area-level socioeconomic factors and all-cause mortality [16]. Other studies, including several studies from this study population of Northern Ireland, find that when individual characteristics are accounted for, socioeconomic measures at the area-level do not increase risk of suicide [17], alcohol-related [8] or injury death [10]. However, many of these studies test effects of general measures of area quality (e.g. material deprivation), which might correlate with individual SEP, and few compare different types of preventable death or explore how effects vary by sex and SEP. To address these concerns, and to offer possible insights into the patterning of area effects, we test predictions from a robust literature in evolutionary biology on managing risk and uncertainty: life history theory (LHT).

## 

### Life History Theory

LHT is a set of principles that concern an organism’s optimal allocation of resources [18, 19]. LHT can provide an ultimate perspective for why individuals in different environments have different incentives to invest effort that may decrease their risk of death [20–22]. Human behaviour has not evolved to maximize individual health or longevity, but genetic fitness. Because resources are finite, and one unit of energy allocated to one function cannot also be allocated elsewhere, individuals face trade-off decisions between, for example, somatic maintenance (i.e. health) and other adaptively relevant functions. A core principle of LHT is that individuals should be sensitive to the local ecological cues that influence optimal behaviour. In harsh environments, where mortality risk is high and resources are scarce, individuals should adopt a ‘faster’ life history strategy, i.e. faster growth, earlier maturation and reproduction, and exhibit higher degrees of future discounting, as payoffs from long-term investments are less likely to arise [18].

Within LHT research, considerable attention has been given to the impact of extrinsic mortality rate (EMR), i.e. mortality that cannot be mitigated by individual action. For example, in a theoretical model Nettle [21] showed that the level of optimal preventative health effort is lower among individuals with high extrinsic mortality risk than those with low extrinsic mortality risk. If individuals with low SEP have higher extrinsic mortality risk, lower allocation to long-term health can be seen as adaptive. It is both theoretically and empirically challenging to isolate extrinsic from intrinsic mortality [23]. Some experimental evidence suggests that higher perceived extrinsic mortality is associated with a lower level of self-reported health effort, and that primes of ‘uncontrollable’ mortality can affect the choice of healthy versus unhealthy food rewards [24, 25]. But sophisticated tests of the effect of the local EMR remain scarce. Many studies rely on crude proxies (e.g. total or child mortality rate) and/or use mortality rate aggregated at a high level (e.g. country level) that do not accurately capture the local ecology. Wilson and Daly [26] demonstrated in their seminal study that Chicago neighbourhoods with lower life expectancy had higher homicide rates. Wilson and Daly excluded homicides in their life expectancy measure to avoid circularity, and used a small unit (neighbourhoods) as the local ecology. However, their study suffers from the limitations that come with use of aggregated data without individual controls. We address these issues by using a novel operationalization of EMR, based on a public health distinction [27] and calculate this rate at the local ward-level. We in turn use individual preventable death as a proxy for low health investment and explore its relationship with EMR.

Additionally, other types of extrinsic risks should influence the level of health investment. Neighbourhood deprivation is a broad measure of local hardship that might be associated with a range of extrinsic risks, and correlates with several life history traits in the UK [28]. It remains unclear whether individuals in more deprived neighbourhoods respond to higher EMR, resource scarcity or to various other ecological cues correlated with deprivation. Studying observable health behaviours (smoking and physical exercise) in two Newcastle neighbourhoods, Nettle [29] found greater differences than would be predicted by the differences in socioeconomic factors between those neighbourhoods. There might be a range of factors that vary between areas of similar levels of deprivation, and have impact on health behaviour. One such factor is local crime rate, which might serve as a cue to risk of losing wealth or incurring health risks from causes beyond individual control. High crime rates might increase perceived environmental threat and uncertainty, which have been associated with a faster life history trajectory [30, 31], thus we test the effect of crime alongside EMR. In our population, specific sub-categories of crime, such as homicides, are too few to study separately and we use an overall measure of crime.

### Adult Sex Ratio

Sexual selection has a role in explaining variation in preventable death. There is substantial evidence that men are more likely to engage in hazardous behaviours and to suffer higher risks of preventable death than women [32, 33]. It has been assumed that such sex differences in preventable mortality, and the fact that (violent) risk-taking behaviours are more prevalent among non-partnered men, arise due to male–male competition for females, either as contest interactions or through competition over resources [33–35]. However, both the theoretical arguments and the empirical evidence that a male-surplus should lead to *a*, more competition, and *b*, more violence, have recently been questioned [36, 37]. Reformulated models of parental investment reject explanations based on fixed gender roles and posit that male and female mating strategies should be sensitive to variation in the adult sex ratio (ASR). Kokko and Jennions [37] and others have argued that males should increase competition when they are in demand, not oversupply, as a higher degree of mating opportunities would select for a strategy to outcompete other males.

This argument is congruent with ‘mating market models’ that suggest that the rarer sex can increase demands on a potential mate, for example, by demanding higher earnings [38]. Higher intrasexual competition does not necessarily lead to higher levels of violence—males might compete non-violently—and which strategy an individual adopts should depend on other factors, such as prowess and resource access [37]. We have previously demonstrated that there is a large sex difference in accidental/suicide deaths (70% higher hazards), and higher preventable death among non-partnered men and men without dependent children in Northern Ireland [39]. Notably, we found that the effects of men’s mating and parenting status were larger for men with low SEP, implying context-dependency of male mating strategies [39]. Here, we build on those findings and test whether a male-biased sex ratio is associated with higher preventable death among men (which would support the ‘more men more violent competition’ model [33]) or lower preventable death (which would support reformulated models). We consider different types of preventable death (see Methodology) and examine the relationship between ASR and preventable death in women.

### Aims Of The Study

We have previously used data from this population to test individual predictions regarding mating and parenting status on preventable death [39], and ecological effects on timing of first birth [40]. We found evidence that a higher EMR, crime and a female-biased sex ratio accelerate motherhood, and that higher EMR and crime rate accelerate fatherhood [40]. Here, we turn to the ecological effects on preventable death and address a number of methodological shortcomings in the previous literature. First, when studying risk or health behaviours, using *extrinsic* mortality rate is crucial to avoid circularity. Using the total population of Northern Ireland, we calculate a ward-level EMR based on deaths deemed unavoidable [27]. Second, the size of the area taken to represent the local ecology is important: too big leads to large variation within areas and too small leads to unstable mortality rates. We use the Northern Irish ward-level (*n*∼2900 individuals, 570 wards), which eschews both these issues. Third, this uniquely detailed database enables us to test for differences with sex and SEP, which are often overlooked in previous literature, but might offer important insights.

We first test a prediction related to the social gradient in health behaviours: that low SEP individuals should have higher risk of extrinsic death [21]. Then, we test predictions that higher EMR and crime rate should be associated with higher preventable death, and examine whether a skewed sex ratio is associated with higher or lower risk of death in the mate-limited sex. Using data on different causes of preventable death enables us to explore whether both death from risk-taking (accidents/suicides and excessive alcohol consumption) and long-term health neglect (other preventable diseases) are responses to local ecologies.

## METHODOLOGY

### Data

The Northern Ireland Mortality Study (NIMS) is a database from Northern Ireland handled by the Northern Ireland Statistics and Research Agency (NISRA). The database comprises all approximately 1.6 million enumerated individuals who resided in Northern Ireland on the 29th of April 2001. Our data comprise all deaths to individuals aged 16–59 years at the Census, that occurred in Northern Ireland between the Census and an 8.7-year follow up period. Note that the period of risk commences after the ‘Troubles’ (c. 1969–1998), when over 3600 people died as a consequence of sectarian conflict between Catholics and Protestants [41]. Although some sporadic localized conflict still exists, the direct effect of the Troubles on preventable causes of death in this sample is likely to be minimal. The lower age restriction was set because socioeconomic data are available only for individuals aged 16 or older, and age was capped at 59 as selection pressures should be stronger for traits exhibited earlier in life and including old individuals might obscure these. Missing data were imputed by the Census [42]. The data are held in a safe setting by NISRA and were made available for this study. Ethical permission was acquired from NIMS.

### Preventable death

The cause of death was recorded by the general practitioner, as International Classifications of Disease codes, 10th edition (ICD-10). Our outcome measures of preventable deaths are based on causes of death deemed ‘avoidable’ according to the classification by Page *et al.* ([27], see also [43]). We take a conservative approach and examine only deaths deemed to be directly preventable by individual behaviour (‘avoidable’), and not deaths that might be avoidable with timely and appropriate medical help or societal action (known as ‘amenable’ causes). This means, for example, that some accidents are considered extrinsic whereas others are considered intrinsic (see Supplementary information). None of the causes of death we examine can be considered entirely extrinsic, but we believe that this definition fits well with the distinction made within LHT. We analyse two separate outcomes (i) accidental/suicide and alcohol-related deaths and (ii) other preventable diseases (where the largest single category is lung cancer). The time between health behaviour and death might sometimes be longer than the 9 years of risk we have data on (e.g. for smoking and lung cancer death). We are interested in the overall patterns of all preventable diseases and should capture some effects of the local ward—if they exist—even with this time frame. Whilst accidents, suicides and alcohol-related deaths are distinct causes, the relatively small population of Northern Ireland in combination with a relatively short time at risk, does not allow for separate analyses of these causes. Examining effects by age, sex and SEP limits sample sizes further. Thus, for models run by SEP, we collapse the outcomes to ‘any preventable death’. For a detailed description of these outcomes, see Supplementary information.

### Independent variables

We calculated EMR per 100 000 (mean 284.9, SD 154.7) based on unpreventable deaths (all causes of death that are not ‘avoidable’ by Page *et al.*rsquo;s definition, (i.e. are either amenable or unavoidable)) to individuals aged 16–74 years, divided by the total ward population (*n*∼2900) of the same age range, multiplied by 100 000. We use data on 570 of the 582 wards, as some wards were very scarcely populated and had to be excluded.

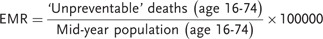

The lower age cap was set at 16 because we are interested in adult mortality rate, and the upper age cap is set at 74, because after this age all deaths are considered to some extent unavoidable [27]. EMR was not highly correlated to the mean age of the ward (*r* = 0.27, *P* < 0.000).

Data on crime were taken from a standardized super output area (SOA) level crime and disorder deprivation, a composite measure of different aspects of crime from NISRA (only available at SOA level), and aggregated at the ward-level. ASR was calculated as the proportion of men:women aged 15–50 for year 2002 (mean 0.98, SD 0.098, see also Supplementary information). We also calculated a sex ratio based on individuals aged 15–39 but found no difference in the results and retained the 15–50 age range. The ecological variables were made into tertiles to allow for easier interpretation and as a way of standardizing variables (tertiles was a better fit than quartiles, quintiles or sixtiles).

All analyses adjust for a range of individual-level variables. This enables us to determine whether predicted area effects are influential above and beyond what can be captured by individual data. The effects of the individual variables included here have previously been described in Ref. [39]. See Supplementary information for distributions of all individual variables (Supplementary Tables S1 and S2). We control for age, marital/cohabiting status, dependent children in the household, economic activity, highest level of education (none, GCSE/1 A-level, 2 or more A-levels, university degree), housing tenure (social housing, privately renting, privately owned) and household car access (none, one, two or more), community background (Catholic, Protestant, none/other) and residence type (Belfast, Derry, towns, rural areas). In models stratified by SEP, we use a SEP index based on highest level of education, housing tenure and car access (see Supplementary information). Low SEP is coded as points on the index scale of 0–3 and high SEP as 4–7. We exclude individuals (*n*∼21 000) who reside in communal establishments (non-households, e.g. care homes, hospitals or prisons) because these individuals lack socioeconomic variables. Ethnicity was not included as less than 0.4% had another ethnicity than ‘white’, most likely a result of very low levels of immigration due to a history of sectarian conflict.

### Statistical analysis

We run Cox proportional hazard models with preventable death as the outcome, separately for men and women (in total, 7 967 318 person-years-at-risk). We tested for clustering at the ward-level (i.e. that two individuals within the same ward are more similar to each other than any two random individuals from the population) by running models with shared frailty (random intercept model for Cox regression). Once individual SEP was controlled for, the random intercept was not significant and the frailty term was removed to achieve a more parsimonious model. The proportionality assumption, that hazards do not change over the study period was checked according to Ref. [44] and fulfilled for all variables apart from age. We therefore ran models separately by age (younger, 16–44, and older, aged 45–59 years in 2001). This cut-off was chosen because most individuals who will ever have children will have had them at age 44. Throughout this paper, we focus on the younger cohort (i.e. deaths between 16 and 54 years) because we are mostly interested in the behaviour of individuals of reproductive age and want to limit deaths from causes that become increasingly unpreventable as individuals age. We present results for the older individuals (deaths between age 45 and 68) in the Supplementary information (Supplementary Tables S3 and S4). All analysis was performed using STATA 12.

### Model selection

We define *a priori* candidate models based on all combinations of the ecological variables (models *a–h*, see below). We rely on Akaike Information Criterion (AIC), to compare model fit. The model with the lowest AIC is the best fitting model for the data. Models can then be weighted on the basis of how much their AIC value increases as compared with the best fitting model (which has an AIC change of 0). A decrease in AIC value of two or more implies a better model fit [45]. EMR denotes ward-level extrinsic mortality rate, CR crime rate and adult sex ratio (ASR). The following candidate models were compared:

*a*, Individual

*b*, Individual + EMR

*c*, Individual + CR

*d*, Individual + ASR

*e*, Individual + EMR + CR

*f*, Individual + EMR + ASR

*g*, Individual + CR + ASR

*h*, Individual + EMR + CR + ASR

## RESULTS

**Figure 1.**
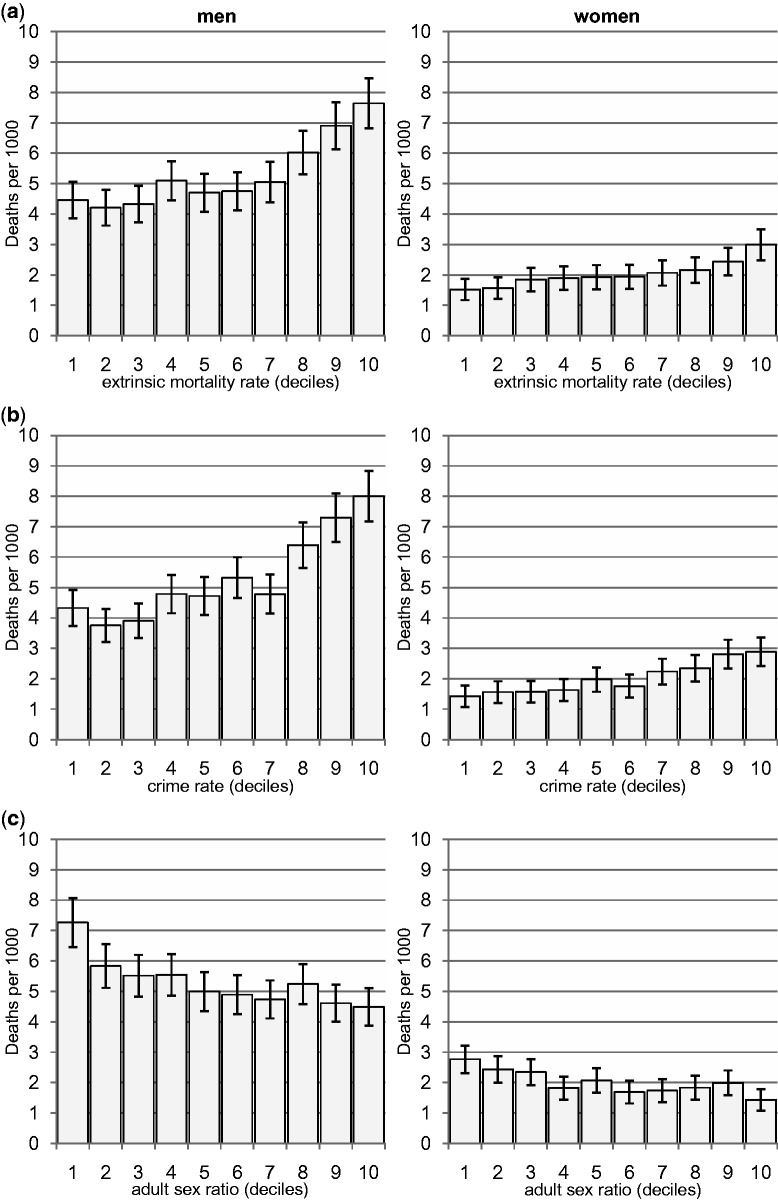
**(a–c) **Accidental/suicide or alcohol-related death for men (*n* = 454 497) and women (*n* = 472 653), by (a) extrinsic mortality rate, (b) crime rate and (c) adult sex ratio

About half (49.8%) of the deaths to individuals aged 16–59 in 2001 are due to extrinsic and half are due to intrinsic causes. Accidental deaths are high among youth, whereas alcohol-related deaths and other preventable deaths show a strong positive relationship with age. The raw data reveal that EMR, crime rate and a female-biased sex ratio are all associated with higher death from accidents resulting from risk-taking behaviour, suicides or alcohol (henceforth, ‘risky’ death) for both men and women (Fig. 1a–c). Death from other preventable diseases follows similar patterns (see Supplementary Figs S1 and S2).

### Is there a socioeconomic gradient in extrinsic death?

**Figure 2.**
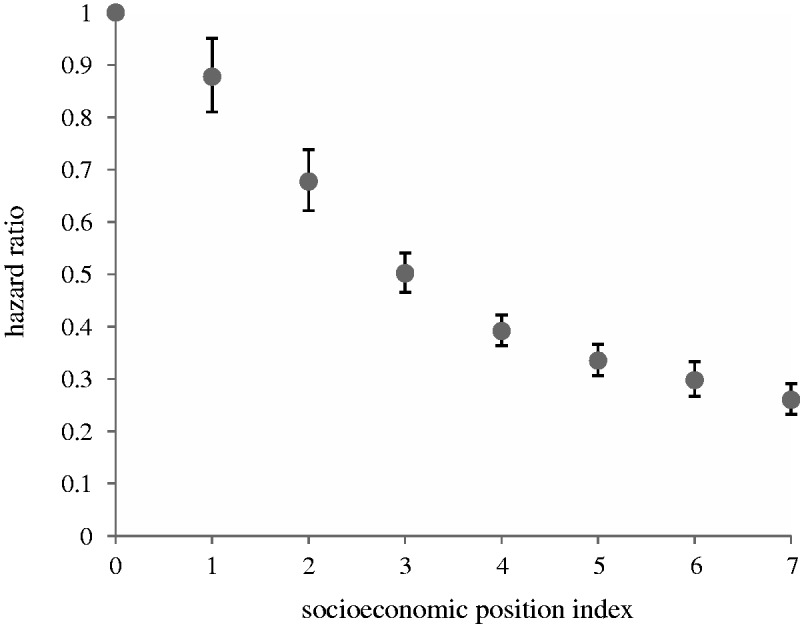
HRs of individual risk of extrinsic death, by SEP (*n* = 927 150). SEP (where 0 is the lowest and 7 the highest) is based on Census variables: highest level of education, housing tenure and household car access. Model also controls for sex, age, marital/cohabiting status, dependent children in the household, community background (Catholic, Protestant, none/other) and residence type (Belfast, Derry, towns, rural areas)

We test whether lower SEP predicts higher risk of extrinsic death (as a dependent variable). Note that in all subsequent analyses EMR is an independent variable predicting preventable death. There was a clear socioeconomic gradient in extrinsic death risk (Fig. 2). Individuals with the highest SEP have 74% lower hazard of extrinsic death compared with those with the lowest SEP.

### Model selection results

We present the model selection results for risky death and other preventable death by sex and SEP. We refer to the direction and magnitude of the effects in the text, but present all effect sizes (Supplementary Tables S5–S8) and hazard ratios (HRs) of individual variables (Supplementary Table S9) in the Supplementary information.

The best model for male risky death is a model with individual variables, EMR and crime (Table 1). Men in the highest EMR tertile have 18% higher hazard than peers in the lowest tertile (HR 1.18 [1.03–1.34]) with a similar effect size for crime rate (HR 1.21 [1.02–1.43]) (see Supplementary Table S5). Effect sizes of both EMR and crime rate are altered only slightly when both variables are in the model as compared with when they are added individually, implying that only a very slight proportion of the effects of EMR is due to the fact that such areas also have higher crime rates (see Supplementary Table S5). Men also have higher risk of other preventable death in wards with high EMR (HR 1.26 [1.05, 1.51]) (Supplementary Table S5). ASR does not appear in any of the best fitting models for male death (Table 1).

**Table 1.** **Men **(aged 16-44 years in 2001)

	Accident/suicide/alcohol-related death(*n* = 317 156, deaths = 1438)				Other preventable diseases (*n* = 317 156, deaths = 735)			
Model	*k*	AIC	ΔAIC	*w_i_*	*k*	AIC	ΔAIC	*w_i_*
Ind	28	35 403.6	6.2	0.02	28	17 961.8	3.8	0.10
Ind+EMR	30	**35 398.3**	**0.9**	**0.30**	30	**17 958.0**	**0.0**	**0.66**
Ind+CR	30	35 400.5	3.1	0.10	30	17 965.5	7.5	0.02
Ind+ASR	30	35 407.5	10.1	0.00	30	17 965.5	7.5	0.02
Ind+EMR+CR	32	**35 397.4**	**0.0**	**0.46**	32	17 962.0	4.0	0.09
Ind+EMR+ASR	32	35 402.2	4.8	0.04	32	17 961.8	3.8	0.10
Ind+CR+ASR	32	35 404.4	7.0	0.01	32	17 969.2	11.2	0.00
Ind+EMR+CR+ASR	34	35 401.3	4.0	0.06	34	17 965.8	7.8	0.01

Model selection results. EMR, extrinsic mortality rate; CR, crime rate; ASR, adult sex ratio; AIC, Akaike information criterion; *w_i_*, Akaike weight. Best fitting model(s) in bold.

Among women, two models better predict risky death than the rest of the candidate models: the individual model has a 53% probability of being the true model, but a model also including ASR has 22% probability of being the true model (Table 2). Women in the most male-biased areas have 17% higher hazard of risky death (Supplementary Table S6). For other preventable diseases, the individual model was the best model among women, with 65% probability of being the true model (Table 2).

**Table 2.** **Women **(aged 16–44 years in 2001)

	Accident/suicide/alcohol-related death (*n* = 331 839, deaths = 507)				Other preventable diseases (*n* = 331 839, deaths = 556)			
Model	*k*	AIC	ΔAIC	*w_i_*	*k*	AIC	ΔAIC	*w_i_*
Ind	28	**12 429.0**	**0.0**	**0.53**	28	**13 616.6**	**0.0**	**0.63**
Ind+EMR	30	12 432.5	3.5	0.09	30	13 620.1	3.5	0.11
Ind+CR	30	12 432.5	3.5	0.09	30	13 619.8	3.3	0.12
Ind+ASR	30	**12 430.8**	**1.8**	**0.22**	30	13 620.5	4.0	0.09
Ind+EMR+CR	32	12 436.4	7.0	0.02	32	13 623.5	6.9	0.02
Ind+EMR+ASR	32	12 434.3	5.3	0.04	32	13 624.1	7.5	0.01
Ind+CR+ASR	32	12 436.4	7.4	0.01	32	13 623.8	7.2	0.02
Ind+EMR+CR+ASR	34	12 440.0	11.0	0.00	34	13 627.5	10.9	0.00

Model selection results. EMR, extrinsic mortality rate; CR, crime rate; ASR, adult sex ratio; AIC, Akaike information criterion; *w_i_*, Akaike weight. Best fitting model(s) in bold.

### Ecological effects by SEP

**Figure 3.**
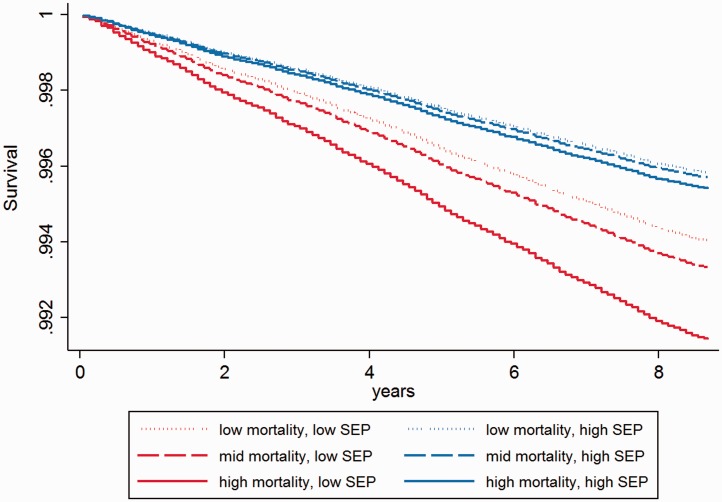
Preventable death by EMR and SEP. Cox proportional hazards survival function for young men (aged 16–44 in 2001) by individual SEP (low SEP (SEP score of 0–3) high SEP (SEP score of 4–7) and EMR (tertiles)). Model also controls for age, marital/cohabiting status, dependent children in household, economic activity, community background (Catholic, Protestant, none/other) and residence type (Belfast, Derry, towns, rural areas)

Model selection performed separately by SEP revealed some differences in the best candidate models. For men, EMR is in the best fitting model among low SEP men, but crime is in the best fitting model for men with high SEP (Table 3 and Supplementary Table S7). Interaction tests revealed that the effect of EMR on any preventable death is greater among men with low SEP than men with high SEP (the decrease in AIC is larger than 2 in the interaction model compared with the main model, see Supplementary Table S10). Figure 3 illustrates this disadvantage for low SEP men in high mortality areas with high EMR. Effect sizes are large: hazards are 39% higher in the highest EMR tertile compared with the lowest (HR 1.39 [1.20, 1.63]) among low SEP men (Supplementary Table S7). Among women, the individual model, followed by Individual + Crime rate are the best fitting models, regardless of SEP (Table 4 and Supplementary Table S8).

**Table 3.** **Preventable death by SEP, men **(aged 16–44 years in 2001)

	Low SEP (*n* = 96 476, deaths = 1139)				High SEP (*n* = 220 680, deaths = 956)			
Model	*k*	AIC	ΔAIC	*w_i_*	*k*	AIC	ΔAIC	*w_i_*
Ind	21	25 534.9	17.9	0.00	21	**23 186.2**	**1.5**	**0.22**
Ind+EMR	23	**25 516.9**	**0.0**	**0.66**	23	23 188.5	3.9	0.07
Ind+CR	23	25 535.4	18.4	0.00	23	**23 184.7**	**0.0**	**0.45**
Ind+ASR	23	25 538.2	21.3	0.00	23	23 188.7	4.0	0.06
Ind+EMR+CR	25	25 519.6	2.7	0.18	25	23 187.9	3.2	0.09
Ind+EMR+ASR	25	25 520.3	3.3	0.13	25	23 191.1	6.4	0.02
Ind+CR+ASR	25	25 538.6	21.6	0.00	25	23 188.2	3.5	0.08
Ind+EMR+CR+ASR	27	25 522.9	6.0	0.03	27	23 191.3	6.7	0.02

Model selection results. EMR, extrinsic mortality rate; CR, crime rate; ASR, adult sex ratio; AIC, Akaike information criterion; *w_i_*, Akaike weight. Best fitting model(s) in bold.

**Table 4.** **Preventable death by SEP, women **(aged 16–44 years in 2001)

	Low SEP (*n* = 103 886, deaths = 543)				High SEP (*n* = 227 953, deaths = 481)			
Model	*k*	AIC	ΔAIC	*w_i_*	*k*	AIC	ΔAIC	*w_i_*
Ind	21	**12 099.6**	**0.0**	**0.41**	21	**11 615.3**	**0.0**	**0.32**
Ind+EMR	23	12 101.5	2.0	0.16	23	11 617.5	2.2	0.11
Ind+CR	23	**12 100.9**	**1.3**	**0.21**	23	**11 615.9**	**0.5**	**0.24**
Ind+ASR	23	12 103.5	3.9	0.06	23	11 617.9	2.5	0.09
Ind+EMR+CR	25	12 102.4	2.9	0.10	25	11 617.6	2.2	0.10
Ind+EMR+ASR	25	12 105.5	5.9	0.02	25	11 620.2	4.8	0.03
Ind+CR+ASR	25	12 104.9	5.3	0.03	25	11 618.1	2.7	0.08
Ind+EMR+CR+ASR	27	12 106.4	6.8	0.01	27	11 620.0	4.6	0.03

Model selection results. EMR, extrinsic mortality rate; CR, crime rate; ASR, adult sex ratio; AIC, Akaike information criterion; *w_i_*, Akaike weight. Best fitting model(s) in bold.

## DISCUSSION

Individuals’ behavioural response to their local environment is a central question in life history research. We have made use of uniquely detailed data from Northern Ireland to test multiple ecological effects on a large number of local areas. Our study is an important contribution to the current literature because most previous studies have not been able to explore multiple local area-level effects whilst controlling for individual factors, nor explored how these effects vary with sex or SEP, or with type of preventable death. We also make a methodological contribution by deploying a novel way of operationalizing *extrinsic* mortality rate. We have previously confirmed findings from studies in developed countries that individual socioeconomic differences account for a high proportion of the variation in preventable death [39], but here demonstrate independent and additive effects of the local area, that vary with sex and SEP. Interestingly, the best model for risky death among males, individual variables, EMR and crime, was also the best model in our analysis if risk of fatherhood in this population [40].

We found support for the prediction that low SEP individuals run higher risks of dying from causes beyond their own control. This underpins the idea that the SEP gradient in health is due to varying fitness returns from health investment [20, 21]. Moreover, we found support for the prediction of higher risk of preventative death in areas with high EMR and crime among men, but not among women. For male death, results indicated that effects of EMR and crime were independent of each other. This highlights the importance of testing multiple factors on life history strategies, rather than considering the overall effect of a ‘harsh’ or ‘deprived’ area. Furthermore, the effect of EMR was driven by low SEP men and suggests that men with more resources might be buffered from local mortality risk by their wealth. This question is complicated by the fact that extrinsic death (as an individual outcome) is correlated with individual SEP. This presents both a methodological and theoretical challenge and begs the question to what degree it is possible to disentangle individual and area-level effects.

We found no evidence of an effect of ASR on either type of preventable death among men. Among women, on the other hand, there was some evidence that a male-biased sex ratio was associated with higher risk of accidental death, suicide and alcohol-related death. At first glance these results might seem odd; when women are in demand, they should not have to resort to hazardous behaviour to secure or retain a mate. It is possible that higher female death in male-biased areas arise if males exert more mate-guarding, in terms of physical or mental coercion towards females who wish to switch partners. A different interpretation, consistent with mating market models, would be that if men in male-biased areas are responding to demands of women, for example, to provide more childcare, women might be free to inhabit more typically male domains that might be associated with higher risks. It is also possible that the women who remain in male-biased areas face various structural inequalities that are associated with poorer health outcomes. It should be noted that death is a crude measure of intra-sexual competition, and other types of measures, e.g. non-lethal violence might better capture sex ratio-related variation in mating behaviour.

An assumption we make is that individuals are sensitive to ward-level cues, or at least that the ward meaningfully represents the local ecology which might have impact on the individual’s somatic state [46]. Whilst we cannot address precise proximate mechanisms, it seems very unlikely there are genetic differences between people in different areas in a well-mixed urban setting with some migration. In modern urban environments people move, travel for work and consume media, which might lessen the influence of their residential environment. Nevertheless, Nettle *et al.* [47] have shown that visitors who spend a very short period of time in a neighbourhood, report a level of social trust similar to that of permanent residents of that neighbourhood. So although people who have recently moved to an area have less time to adapt to those conditions, it need not impair ability to pick up local cues.

Moreover, different cues of the local ecology might vary in how they affect behaviour. One does not have to pick a mate from within one’s residential ward, but it might be difficult to escape effects of local crime. However, it could be argued that even if it is possible to choose a mate from another ward, some aspect of living in an area with scarcity of mates might influence behaviour. Indeed, ward-level ASR has substantial effect on age at first birth in Northern Ireland, implying that the ASR or some related factor affects mating behaviour [40]. The relevance of the ward might vary with urban versus rural residence, or with SEP, as rural residents and low SEP individuals might be more constrained to interactions nearby. This could potentially explain why ecological measures had stronger effects among low SEP men in our analyses. Whether individuals’ perceptions of their local environment map on to real conditions, and the role of unpredictability of ecological conditions independently of degree of area quality, should be explored in future research.

Life history studies have often focused on either reproductive or risk-taking behaviours, but here we apply similar predictions to examine preventable death. The presented results corroborate the idea that investing effort in health and avoiding risks are not necessarily adaptive strategies in environments where risk of extrinsic mortality and crime are high. This is an important insight for policy makers shaping campaigns of safe drinking, driving and other health-related behaviours. Policy makers should be aware that individual SEP has large effects on preventable death but also that area-level characteristics such as higher EMR and crime are associated with higher preventable death, especially for men. Reducing crime might be as important as lowering mortality rates, but both factors are likely to have positive effects on a range of health behaviours.

## SUPPLEMENTARY DATA

Supplementary data is available at *EMPH *online.

Supplementary Data

Supplementary Data
