# Supplementary material for: Effects of local extrinsic mortality rate, crime and sex ratio on preventable death in Northern Ireland
Source: Evol Med Public Health. 2015 Sep 3;2015(1):266–77. doi: 10.1093/emph/eov020 (PMC4604479; doi:10.1093/emph/eov020)
Supplement: Supplementary Data [file supp_2015_1_266__index.html]

Effects of local extrinsic mortality rate, crime and sex ratio on preventable death in Northern Ireland — Supplementary Data 

# Effects of local extrinsic mortality rate, crime and sex ratio on preventable death in Northern Ireland

## Supplementary Data

files

- Supplementary Data - docx file
