# Supplementary material for: Effects of local extrinsic mortality rate, crime and sex ratio on preventable death in Northern Ireland
Source: Evol Med Public Health. 2015 Sep 3;2015(1):266–77. doi: 10.1093/emph/eov020 (PMC4604479; doi:10.1093/emph/eov020)
Supplement: Supplementary Data [file supp_eov020_150812_SI_EMPH.docx]

Supplementary Information

In this paper, we deploy a definition of different causes of mortality, following Page et al. 2006. Page et al. divide all potential causes of death into preventable and unavoidable as follows:

- Preventable (‘*avoidable*’, can be avoided by individual behaviour OR ‘*amenable*’ to health care or societal action).
- Unavoidable (neither amenable to health care, or avoidable through individual behaviour).

We take a conservative approach and use only the deaths considered ‘avoidable’ by Page et al. as preventable/intrinsic mortality (1, see also 2). This is because from a life history point of view, we are interested in those causes of death that deemed to be almost completely within individual influence. Including amenable deaths is not desirable as we cannot be sure that the death could have been avoidable through individual action. Note that the number of unavoidable deaths is very low in Northern Ireland, hence why extrinsic mortality rate (EMR) is based on all deaths that are NOT considered ‘avoidable’ by Page et al. 2006 (that is amenable + unavoidable).

Preventable death outcomes

Accidents/suicides

Deaths in this category include falls, certain traffic accidents, suicides, accidental deaths with ‘undetermined intent’ (potential suicides), other accidents, violence, homicide, or exposure to drugs. Although suicides and accidental deaths are distinct causes, they are collapsed here, because they share similarities such as being instantaneous, and also because they are sometimes difficult distinguish between (e.g. “undetermined intent”). Note that the distinctions are very detailed; some traffic accidents are considered extrinsic (e.g. a cyclist dying in a collision with a lorry) whereas a driver who is in a collision with a stationary object is considered as an intrinsic/preventable death. Of the category accidents/suicides (n=2146) accidental deaths are responsible for 1256 deaths, and 890 are suicides. Our definition differs slightly from the Page et al (1) version of ‘avoidable’ as we decided to also include c. 190 causes of death, e.g. V.43 and V.44 car traffic accident & with heavy (n~86), accidental poisoning (X.44, n~37) along with some other causes that cannot be revealed due them being rare and thus violate anonymity according to the Northern Ireland Longitudinal Study regulations.

Alcohol-related causes of death

The majority of this group consists of liver related diseases (cirrhosis of the liver) caused by excessive alcohol consumption. Accidents where alcohol might have been a contributing cause (*n*=43) are included as outcomes both in accidental deaths and suicide deaths.

Other preventable diseases

These are all other medical causes that are deemed preventable by individual action. Among the other diseases (n=5907), the single largest category is lung cancer deaths (n=1572), other preventable cancers (n~2400), diseases of the circulatory system (n~365), diseases of the respiratory system (n~500), and diseases of the digestive system (n~250) also contributing. Because some causes might be less than 10, and thus classified as disclosive, the numbers given here are not exact in order to protect anonymity.

Total preventable deaths

For the total preventable death outcomes, we adhere to the Page et al. (1) definition of ‘avoidable’ deaths for sake of comparability. This allows us to be more specific and tailor the specific sub-categories of death to a definition closer to what we consider is likely to be “extrinsic”, whilst also adhering to the public health defining for total preventable causes. This means that the difference in total preventable death (*n*=9,075) is slightly lower than the sum of sub-categories of death but the difference is very small in absolute terms and accounts for 0.02% of preventable deaths being classified in a different manner. We wish to include as many individuals as possible, whilst limiting the causes of death that become increasingly unpreventable as people age (e.g. deaths from falls and most cancers). We therefore include deaths to individuals up 59 in 2001 and therefore a maximum age of 68 years at death in 2009).

Independent variables

Extrinsic mortality rate (EMR)

The extrinsic mortality rate is based on causes of death considered amenable and unavoidable as defined by Page et al. We use age range 16-74 since it is adult death rate that should govern life history strategies (child mortality is very low in Northern Ireland). After 74 years of age all causes of death are considered to some extent unavoidable. Age specific extrinsic mortality rates were calculated on ward-level for year 2002, e.g. the first full year following the 2001 Census. Northern Ireland is divided into 580 wards, and 890 Super output areas (SOAs). The lower level SOA, is sometimes the same as a ward if the SOA is large (323 small wards comprise only one SOA). We use ward to avoid too unstable estimates caused by small sample sizes. We did not calculate sex-specific extrinsic mortality rates as these too would be too small. We were unable calculate mortality rates for 9 wards (located along the north coast) since these are very scarcely populated and no deaths occurred here in 2002. It is important to point out that moving between wards is fairly low. 91.9% of people in the current sample (16-59 in 2001, *n*=927,150) lived in the same ward one year before the 2001 Census, and 95.8% lived in the same district one year before the Census, and if considering only those aged 30 or above (*n*=631,197) 94.4% lived within the same ward and 97.2% within the same district.

Adult sex ratio (ASR)

The ASR ranges were as follows: lowest tertile 0.65-0.939, middle tertile 0.94-1.00, and highest tertile 1.01-1.61. All ten wards that had an ASR over 1.2 were wards that contained and SOA with an army base in 2001. We tested models with and without those army SOAs, but found no difference in the results. An extremely low number of preventable deaths occurred in any of those wards (exact figure cannot be given for disclosure reasons).

Individual level independent variables

For marital status we combine marital status and current living arrangements, as to measure cohabitation with partner rather than just marital status. Dependent offspring are children (aged 18 or younger) who reside in the household at the time of the 2001 Census. We include data on a number of different socioeconomic variables: household car access (0, 1 or 2 or more), housing tenure (social housing, privately rented or privately owned), education (no education, lower secondary (1 A level or GCSEs), upper secondary (2 A-levels or more), or university degree. We also include economic activity at Census (active, unemployed, student, retired, home maker, permanently ill, other) since it was a better fit than socioeconomic class, and better reflects current resource access. For models run separately by SEP, we created an index based on tenure, car access and highest level of education, where each increasing level of SEP corresponds to one point, rendering a scale of increasing SEP from 0 to 7. For example, low SEP might refer to an individual who has GCSE/1 A-level (1p), access to one car (1p), and lives in social housing (0p), or an individual who has 2 A-levels (3p), resides in social housing (0p), has no car access (0p).

References

1. Page A, Tobias M, Glover J, Wright C, Hetzel D, Fisher E. Australian and New Zealand Atlas of Avoidable Mortality. Adelaide: University of Adelaide:PHIDU; 2006.

2. Wheller L, Baker A, Griffiths C, Rooney C, Davy M, Jones J. Trends in avoidable mortality in England and Wales 1993-2005. Heal Stat Q. 2007;34:6–25.

Figure S1a-c. Other preventable diseases for men (n=454,497) and women (n=472,653), by (a) extrinsic mortality rate, (b) crime rate, and (c) adult sex ratio.

| Table S1. Descriptive statistics, men. Percentage of total population, and deaths by independent variables. | | | | | | | |
| --- | --- | --- | --- | --- | --- | --- | --- |
|  |  | MEN | | | | | |
|  | | **Total,**  ***n*=454,497** | **Accidents**  ***n*=1,609** | **Alcohol**  ***n*=827** | **Other**  ***n*=3,635** | **All prev.**  ***n=*5,914** | **All deaths**  ***n*=10,793** |
| **Age** | 16-19 | 10.5% | 11.8% | 2.7% | 1.5% | 3.8% | 2.6% |
|  | 20-24 | 10.7% | 10.8% |  | 1.5% | 3.7% | 2.5% |
|  | 25-29 | 11.1% | 10.0% |  | 1.8% | 3.9% | 2.8% |
|  | 30-34 | 12.5% | 10.8% | 8.3% | 2.3% | 5.3% | 4.2% |
|  | 35-39 | 13.0% | 12.4% | 14.2% | 4.8% | 8.0% | 7.0% |
|  | 40-44 | 12.0% | 13.3% | 16.4% | 8.4% | 10.7% | 9.7% |
|  | 45-49 | 10.7% | 11.1% | 19.7% | 14.6% | 14.4% | 15.2% |
|  | 50-54 | 10.3% | 10.3% | 22.4% | 24.7% | 20.8% | 22.3% |
|  | 55-59 | 9.3% | 9.5% | 16.3% | 40.4% | 29.4% | 33.7% |
| **Marital status** | Single | 37.6% | 46.2% | 29.3% | 19.2% | 27.2% | 25.4% |
|  | Married/cohabiting | 56.6% | 42.0% | 40.5% | 66.2% | 56.6% | 58.4% |
|  | Sep/div/widowed | 5.8% | 11.8% | 30.2% | 14.7% | 16.2% | 16.3% |
| **Dependent children in household** | None (& age<=44) | 15.7% | 18.2% | 17.8% | 5.6% | 10.4% | 8.8% |
|  | 1 child or more > | 37.3% | 28.1% | 20.3% | 27.9% | 26.9% | 26.5% |
|  | No child (& age>=45) | 28.8% | 30.9% | 14.2% | 8.7% | 15.0% | 12.5% |
|  | Cores. with parents | 18.3% | 22.8% | 47.8% | 57.8% | 47.8% | 52.3% |
| **Education** | University degree | 17.6% | 7.6% | 10.3% | 11.0% | 10.1% | 10.0% |
|  | 2+ A-levels | 9.2% | 6.4% | 4.1% | 4.5% | 4.9% | 4.6% |
|  | 1 A-level/GCSEs | 35.4% | 33.8% | 22.1% | 19.0% | 23.1% | 20.8% |
|  | None | 37.9% | 52.2% | 63.5% | 65.6% | 62.0% | 64.6% |
| **Economic activity** | Active | 71.2% | 53.1% | 31.8% | 47.7% | 46.8% | 45.4% |
|  | Unemployed | 6.3% | 9.9% | 14.6% | 6.5% | 8.4% | 7.8% |
|  | Student | 8.6% | 5.5% |  | 1.3% | 2.2% | 1.6% |
|  | Homemaker | 1.6% | 2.7% | 2.8% | 2.6% | 2.6% | 2.5% |
|  | Permanently ill | 8.0% | 20.5% | 36.9% | 32.7% | 30.4% | 33.1% |
|  | Retired | 1.1% | 0.8% | 3.3% | 3.8% | 2.9% | 3.3% |
|  | Other inactive | 3.2% | 7.6% | 10.6% | 5.5% | 6.6% | 6.5% |
| **Tenure** | Owner occupied | 79.1% | 63.0% | 45.8% | 67.1% | 63.1% | 63.6% |
|  | Privately rented | 7.0% | 8.8% | 8.2% | 6.3% | 7.1% | 6.7% |
|  | Social housing | 14.0% | 28.2% | 46.0% | 26.6% | 29.7% | 29.8% |
| **Car access** | 2 or > | 12.6% | 28.1% | 53.9% | 24.6% | 29.5% | 28.5% |
|  | 1 | 40.1% | 40.5% | 31.2% | 41.9% | 40.4% | 41.0% |
|  | 0 | 47.3% | 31.5% | 14.9% | 33.5% | 30.1% | 30.5% |
| **Religion** | Catholic | 43.4% | 48.7% | 43.5% | 41.1% | 43.4% | 41.9% |
|  | Protestant | 53.9% | 49.2% | 54.7% | 56.5% | 54.4% | 56.0% |
|  | None/other | 2.7% | 2.2% | 1.8% | 2.4% | 2.3% | 2.1% |
| **Residence** | Belfast | 33.0% | 31.6% | 42.6% | 36.5% | 36.2% | 36.3% |
|  | Derry | 5.2% | 4.0% | 6.8% | 5.8% | 5.5% | 5.3% |
|  | Town | 25.2% | 26.9% | 30.0% | 27.2% | 27.5% | 26.9% |
|  | Rural areas | 36.6% | 37.5% | 20.7% | 30.5% | 30.7% | 31.5% |

| Table S2. Descriptive statistics, women. Percentage of total population, and deaths by independent variables. | | | | | | | |
| --- | --- | --- | --- | --- | --- | --- | --- |
|  |  | WOMEN | | | | | |
|  |  | **Total**  ***n*=469,492** | **Accidents**  ***n*=537** | **Alcohol**  ***n*=435** | **Other**  ***n*=2,272** | **All prev.**  ***n*=3161** | **All deaths**  ***n*=7,442** |
|  |  |  |  |  |  |  |  |
| **Age** | 16-19 | 9.8% | 8.2% | 3.9% | 1.1% | 2.0% | 1.3% |
|  | 20-24 | 10.4% | 6.5% |  | 1.7% | 2.3% | 1.5% |
|  | 25-29 | 11.3% | 8.2% |  | 2.2% | 3.0% | 2.4% |
|  | 30-34 | 13.1% | 13.2% | 5.8% | 3.9% | 5.5% | 4.5% |
|  | 35-39 | 13.3% | 13.0% | 11.7% | 5.2% | 7.4% | 7.1% |
|  | 40-44 | 12.2% | 11.9% | 21.8% | 10.4% | 12.2% | 11.4% |
|  | 45-49 | 10.3% | 14.3% | 22.1% | 14.3% | 15.4% | 15.6% |
|  | 50-54 | 10.2% | 13.8% | 18.6% | 24.8% | 22.4% | 23.2% |
|  | 55-59 | 9.3% | 10.8% | 16.1% | 36.5% | 29.8% | 33.0% |
| **Marital status** | Single | 31.3% | 29.1% | 15.4% | 15.1% | 17.2% | 16.3% |
|  | Married/cohabiting | 57.6% | 46.0% | 50.0% | 64.0% | 59.2% | 60.7% |
|  | Sep/div/widowed | 11.0% | 25.0% | 35.0% | 20.9% | 23.6% | 23.0% |
| **Dependent children in household** | None (& age<=44) | 13.8% | 13.6% | 14.0% | 5.2% | 7.8% | 6.1% |
|  | 1 child or more > | 46.3% | 41.3% | 35.0% | 29.9% | 32.1% | 31.9% |
|  | No child (& age>=45) | 19.8% | 15.1% | 5.0% | 6.8% | 7.8% | 7.2% |
|  | Cores. with parents | 20.1% | 30.0% | 46.0% | 58.1% | 52.4% | 54.8% |
| **Education** | University degree | 17.1% | 7.6% | 6.0% | 8.5% | 8.0% | 8.8% |
|  | 2+ A-levels | 11.0% | 5.6% | 4.4% | 4.6% | 4.7% | 4.3% |
|  | 1 A-level/GCSEs | 40.5% | 38.9% | 31.0% | 24.2% | 27.1% | 25.5% |
|  | None | 31.4% | 47.9% | 58.6% | 62.7% | 60.2% | 61.4% |
| **Economic activity** | Active | 57.5% | 35.9% | 29.4% | 34.9% | 34.0% | 34.5% |
|  | Unemployed | 3.1% | 4.5% | 6.2% | 2.7% | 3.4% | 2.8% |
|  | Student | 10.1% | 6.0% |  | 1.1% | 1.7% | 1.2% |
|  | Homemaker | 15.0% | 16.8% | 19.3% | 15.1% | 16.1% | 14.9% |
|  | Permanently ill | 8.4% | 26.1% | 34.7% | 33.5% | 32.7% | 34.6% |
|  | Retired | 1.0% | 10.8% | 10.4% | 4.0% | 3.2% | 3.3% |
|  | Other inactive | 5.0% |  |  | 8.7% | 8.9% | 8.7% |
| **Tenure** | Owner occupied | 75.6% | 58.1% | 49.9% | 64.8% | 61.4% | 64.3% |
|  | Privately rented | 7.9% | 12.1% | 9.2% | 6.1% | 7.6% | 5.9% |
|  | Social housing | 16.5% | 29.8% | 40.9% | 29.1% | 31.0% | 29.8% |
| **Car access** | 2 or > | 16.1% | 33.2% | 44.6% | 26.1% | 30.1% | 27.6% |
|  | 1 | 40.7% | 40.0% | 37.7% | 43.3% | 41.9% | 42.6% |
|  | 0 | 43.2% | 26.8% | 17.7% | 30.6% | 28.0% | 29.8% |
| **Religion** | Catholic | 44.9% | 46.4% | 43.7% | 43.1% | 43.5% | 43.1% |
|  | Protestant | 53.0% | 50.5% | 56.3% | 55.8% | 55.0% | 55.7% |
|  | None/other | 2.1% | 3.2% |  | 1.1% | 1.5% | 1.2% |
| **Residence** | Belfast | 34.8% | 36.7% | 45.1% | 38.6% | 39.6% | 37.6% |
|  | Derry | 5.5% | 5.2% | 5.8% | 6.9% | 6.5% | 5.8% |
|  | Town | 25.9% | 28.3% | 30.3% | 25.2% | 26.3% | 26.8% |
|  | Rural areas | 33.9% | 29.8% | 18.9% | 29.3% | 27.7% | 29.8% |

| **Table S3**. Model selection results, **men** (**aged 45-59 years in 2001**). EMR- extrinsic mortality rate, CR- crime rate, ASR- adult sex ratio. Models also control for age, marital/cohabiting status, dependent children in the household, education, economic activity, household car access, housing tenure, religion, residence type. AIC Akaike information criterion, wi- proportion. Best fitting model(s) (ΔAIC 2 or <) in bold. | | | | | | | | |
| --- | --- | --- | --- | --- | --- | --- | --- | --- |
|  | ***Accident/suicide/alcohol-related death***  (n=137333, deaths=967) | | | | ***Other preventable diseases***  (n=137333, deaths=2897) | | | |
| Model | k | AIC | ΔAIC | w_i_ | k | AIC | ΔAIC | w_i_ |
| Ind | 24 | **21948.2** | **0.0** | **0.36** | 24 | 66873.2 | 16.0 | 0.00 |
| Ind+EMR | 26 | 21950.3 | 2.2 | 0.12 | 26 | 66874.7 | 17.5 | 0.00 |
| Ind+CR | 26 | **21948.5** | **0.4** | **0.30** | 26 | **66857.2** | **0.0** | **0.65** |
| Ind+ASR | 26 | 21952.1 | 4.0 | 0.05 | 26 | 66874.2 | 17.1 | 0.00 |
| Ind+EMR+CR | 28 | 21951.0 | 2.9 | 0.09 | 28 | 66856.0 | 2.8 | 0.16 |
| Ind+EMR+ASR | 28 | 21954.2 | 6.1 | 0.02 | 28 | 66877.0 | 18.9 | 0.00 |
| Ind+CR+ASR | 28 | 21952.4 | 4.3 | 0.04 | 28 | 66856.0 | 2.8 | 0.16 |
| Ind+EMR+CR+ASR | 30 | 21954.9 | 6.8 | 0.01 | 30 | 66863.0 | 5.8 | 0.04 |

| **Table S4.** Model selection results, **women (aged 45-59 years in 2001).** EMR- extrinsic mortality rate, CR- crime rate, ASR- adult sex ratio. Models also control for age, marital/cohabiting status, dependent children in the household, education, economic activity, household car access, housing tenure, religion, residence type. AIC Akaike information criterion, wi- proportion. Best fitting model(s) (ΔAIC 2 or <) in bold. | | | | | | | | |
| --- | --- | --- | --- | --- | --- | --- | --- | --- |
|  | **Accident/suicide/alcohol-related death**  (n=140806,deaths=452) | | | | **Other preventable diseases**  (n=140806, deaths=1713) | | | |
| Model | k | AIC | ΔAIC | w_i_ | k | AIC | ΔAIC | w_i_ |
| Ind | 24 | **10375.3** | **0.0** | **0.63** | 24 | 39683.4 | 2.5 | 0.15 |
| Ind+EMR | 26 | 10379.1 | 3.8 | 0.10 | 26 | **39680.9** | **0.0** | **0.54** |
| Ind+CR | 26 | 10378.3 | 2.9 | 0.15 | 26 | 39685.3 | 4.5 | 0.06 |
| Ind+ASR | 26 | 10381.0 | 5.6 | 0.04 | 26 | 39687.2 | 6.3 | 0.02 |
| Ind+EMR+CR | 28 | 10382.4 | 7.0 | 0.02 | 28 | 39683.8 | 2.9 | 0.12 |
| Ind+EMR+ASR | 28 | 10380.7 | 5.4 | 0.04 | 28 | 39684.7 | 3.9 | 0.08 |
| Ind+CR+ASR | 28 | 10382.0 | 6.7 | 0.02 | 28 | 39689.0 | 8.2 | 0.01 |
| Ind+EMR+CR+ASR | 30 | 10384.1 | 8.8 | 0.01 | 30 | 39687.6 | 6.8 | 0.02 |

|  |  | Accident/suicide/alcohol  Pr3 | | Other preventable death  Other | |
| --- | --- | --- | --- | --- | --- |
|  |  | Young | Old | Young | Old |
| Ind+EMR | 2^nd^ tertile | 1.04(0.91,1.20) | 1.12(0.95,1.32) | 1.04(0.86,1.25) | 0.99(0.90,1.09) |
|  | 3^rd^ tertile | 1.21(1.06,1.38) | 1.05(0.90,1.24) | 1.26(1.05,1.51) | 1.06(0.97,1.16) |
| Ind+CR | 2^nd^ tertile | 1.15(0.99,1.33) | 1.10(0.92,1.33) | 1.03(0.84,1.27) | 1.15(1.04,1.28) |
|  | 3^rd^ tertile | 1.25(1.05,1.48) | 1.22(0.99,1.49) | 1.07(0.85,1.34) | 1.30(1.16,1.45) |
| Ind+AS | 2^nd^ tertile | 0.98(0.86,1.13) | 0.99(0.84,1.16) | 0.95(0.79,1.15) | 0.93(0.85,1.02) |
|  | 3^rd^ tertile | 0.98(0.84,1.16) | 1.00(0.83,1.22) | 0.98(0.78,1.23) | 0.92(0.82,1.03) |
| Ind+EMR+CR | 2^nd^ tertile | 1.03(0.90,1.18) | 1.10(0.94,1.30) | 1.03(0.85,1.25) | 0.97(0.89,1.07) |
|  | 3^rd^ tertile | 1.18(1.03,1.34) | 1.03(0.87,1.21) | 1.26(1.05,1.51) | 1.02(0.93,1.12) |
|  | 2^nd^ tertile | 1.13(0.98,1.31) | 1.10(0.92,1.33) | 1.00(0.82,1.23) | 1.15(1.04,1.27) |
|  | 3^rd^ tertile | 1.21(1.02,1.43) | 1.21(0.98,1.49) | 1.02(0.81,1.28) | 1.29(1.14,1.45) |
| Ind+EMR+AS | 2^nd^ tertile | 1.05(0.91,1.20) | 1.12(0.95,1.32) | 1.04(0.86,1.26) | 1.00(0.91,1.10) |
|  | 3^rd^ tertile | 1.21(1.06,1.38) | 1.06(0.90,1.24) | 1.26(1.05,1.51) | 1.06(0.85,1.03) |
|  | 2^nd^ tertile | 0.99(0.86,1.13) | 0.98(0.83,1.15) | 0.96(0.80,1.16) | 0.93(0.85,1.03) |
|  | 3^rd^ tertile | 0.98(0.83,1.16) | 0.99(0.82,1.20) | 0.98(0.78,1.23) | 0.92(0.82,1.03) |
| Ind+CR+AS | 2^nd^ tertile | 1.16(0.996,1.34) | 1.11(0.92,1.34) | 1.03(0.84,1.27) | 1.15(1.04,1.28) |
|  | 3^rd^ tertile | 1.26(1.06,1.49) | 1.22(0.99,1.50) | 1.07(0.85,1.34) | 1.29(1.14,1.44) |
|  | 2^nd^ tertile | 1.00(0.87,1.15) | 1.00(0.85,1.18) | 0.96(0.79,1.15) | 0.95(0.87,1.04) |
|  | 3^rd^ tertile | 1.02(0.87,1.21) | 1.03(0.85,1.26) | 0.99(0.78,1.24) | 0.96(0.86,1.07) |
| Ind+EMR+CR+AS | 2^nd^ tertile | 1.03(0.90,1.18) | 1.10(0.93,1.30) | 1.04(0.86,1.25) | 0.98(0.89,1.07) |
|  | 3^rd^ tertile | 1.18(1.03,1.34) | 1.03(0.87,1.21) | 1.26(1.05,1.51) | 1.02(0.93,1.12) |
|  | 2^nd^ tertile | 1.13(0.98,1.32) | 1.11(0.92,1.34) | 1.00(0.82,1.23) | 1.15(1.03,1.27) |
|  | 3^rd^ tertile | 1.21(1.02,1.44) | 1.21(0.98,1.49) | 1.01(0.80,1.28) | 1.28(1.14,1.44) |
|  | 2^nd^ tertile | 1.00(0.87,1.15) | 0.99(0.84,1.17) | 0.96(0.80,1.16) | 0.95(0.87,1.05) |
|  | 3^rd^ tertile | 1.01(0.86,1.20) | 1.02(0.84,1.24) | 0.98(0.78,1.24) | 0.96(0.86,1.08) |

Table S5. Cox regressions for male accident/suicide/alcohol-related death and other preventable disease death, by separately by young and old (aged 16-44 and 45-59 years respectively, in 2001). EMR denotes extrinsic mortality rate, CR-crime rate and AS- adult sex ratio. Models also control for age, marital/cohabiting status, dependent children in the household, education, economic activity, household car access, housing tenure, community background and residence type.

|  |  | Accident/suicide/alcohol  Pr3 | | Other preventable death  Other | |
| --- | --- | --- | --- | --- | --- |
|  |  | Young | Old | young | old |
| Ind+EMR | 2^nd^ tertile | 1.07(0.86,1.33) | 1.00(0.78,1.28) | 0.93(0.76,1.15) | 1.09(0.97,1.24) |
|  | 3^rd^ tertile | 1.00(0.81,1.25) | 1.16(0.92,1.47) | 0.95(0.77,1.17) | 1.17(1.04,1.32) |
| Ind+CR | 2^nd^ tertile | 0.93(0.72,1.20) | 1.14(0.86,1.50) | 0.99(0.78,1.25) | 1.06(0.93,1.22) |
|  | 3^rd^ tertile | 1.00(0.76,1.31) | 1.16(0.86,1.56) | 0.91(0.70,1.18) | 1.11(0.96,1.29) |
| Ind+AS | 2^nd^ tertile | 0.97(0.77,1.21) | 0.98(0.78,1.24) | 1.01(0.82,1.25) | 0.98(0.87,1.10) |
|  | 3^rd^ tertile | 1.17(0.89,1.53) | 0.92(0.68,1.23) | 0.99(0.76,1.29) | 1.01(0.87,1.17) |
| Ind+EMR+CR | 2^nd^ tertile | 1.07(0.86,1.34) | 1.00(0.78,1.28) | 0.94(0.76,1.16) | 1.09(0.96,1.23) |
|  | 3^rd^ tertile | 1.00(0.80,1.25) | 1.14(0.90,1.45) | 0.96(0.78,1.18) | 1.16(1.02,1.31) |
|  | 2^nd^ tertile | 0.93(0.72,1.21) | 1.12(0.85,1.47) | 0.99(0.79,1.25) | 1.05(0.92,1.20) |
|  | 3^rd^ tertile | 1.00(0.75,1.31) | 1.13(0.83,1.52) | 0.92(0.71,1.19) | 1.08(0.93,1.26) |
| Ind+EMR+AS | 2^nd^ tertile | 1.07(0.85,1.33) | 1.01(0.79,1.29) | 0.93(0.76,1.15) | 1.09(0.96,1.24) |
|  | 3^rd^ tertile | 1.00(0.80,1.24) | 1.16(0.92,1.47) | 0.95(0.77,1.17) | 1.17(1.04,1.32) |
|  | 2^nd^ tertile | 0.96(0.77,1.21) | 1.00(0.79,1.25) | 1.02(0.82,1.26) | 0.98(0.87,1.11) |
|  | 3^rd^ tertile | 1.16(0.89,1.53) | 0.92(0.69,1.23) | 1.00(0.77,1.31) | 1.00(0.86,1.16) |
| Ind+CR+AS | 2^nd^ tertile | 0.96(0.74,1.24) | 1.13(0.86,1.49) | 0.99(0.78,1.24) | 1.07(0.93,1.22) |
|  | 3^rd^ tertile | 1.02(0.77,1.34) | 1.15(0.85,1.55) | 0.91(0.70,1.18) | 1.12(0.96,1.30) |
|  | 2^nd^ tertile | 0.97(0.78,1.22) | 0.99(0.79,1.25) | 1.00(0.81,1.24) | 0.99(0.88,1.11) |
|  | 3^rd^ tertile | 1.17(0.89,1.54) | 0.94(0.70,1.26) | 0.98(0.75,1.28) | 1.02(0.88,1.19) |
| Ind+EMR+CR+AS | 2^nd^ tertile | 1.06(0.85,1.32) | 1.00(0.78,1.28) | 0.91(0.76,1.16) | 1.09(0.96,1.23) |
|  | 3^rd^ tertile | 0.99(0.79,1.24) | 1.15(0.91,1.46) | 0.96(0.78,1.19) | 1.16(1.02,1.31) |
|  | 2^nd^ tertile | 0.96(0.74,1.24) | 1.11(0.84,1.46) | 0.99(0.79,1.25) | 1.05(0.92,1.21) |
|  | 3^rd^ tertile | 1.02(0.77,1.35) | 1.12(0.82,1.51) | 0.92(0.70,1.20) | 1.08(0.93,1.26) |
|  | 2^nd^ tertile | 0.97(0.77,1.21) | 1.00(0.79,1.26) | 1.01(0.81,1.25) | 0.99(0.87,1.11) |
|  | 3^rd^ tertile | 1.17(0.88,1.54) | 0.94(0.70,1.26) | 0.99(0.75,1.29) | 1.01(0.87,1.18) |

Table S6. Cox regressions for female accident/suicide/alcohol-related death and other preventable disease death, by separately by young and old (aged 16-44 and 45-59 years respectively, in 2001). EMR denotes extrinsic mortality rate, CR-crime rate and AS- adult sex ratio. Models also control for age, marital/cohabiting status, dependent children in the household, education, economic activity, household car access, housing tenure, community background and residence type.

|  |  | Preventable deaths | | | |
| --- | --- | --- | --- | --- | --- |
|  |  | Young  Pr3 | | Old  Pr3 | |
|  |  | Low SEP | High SEP | Low SEP | High SEP |
| Ind+EMR | 2^nd^ tertile | 1.11(0.94,1.32) | 1.03(0.88,1.20) | 1.05(0.94,1.18) | 1.00(0.89,1.13) |
|  | 3^rd^ tertile | 1.40(1.20,1.63) | 1.10(0.94,1.29) | 1.14(1.03,1.27) | 1.01(0.89,1.14) |
| Ind+CR | 2^nd^ tertile | 1.12(0.93,1.35) | 1.14(0.97,1.33) | 1.24(1.08,1.41) | 1.12(0.99,1.28) |
|  | 3^rd^ tertile | 1.21(0.99,1.48) | 1.25(1.03,1.51) | 1.49(1.29,1.71) | 1.19(1.02,1.38) |
| Ind+AS | 2^nd^ tertile | 0.95(0.82,1.11) | 0.93(0.78,1.10) | 0.93(0.84,1.04) | 0.90(0.79,1.03) |
|  | 3^rd^ tertile | 1.02(0.85,1.22) | 0.88(0.72,1.08) | 0.98(0.86,1.11) | 0.85(0.72,0.99) |
| Ind+EMR+CR | 2^nd^ tertile | 1.11(0.94,1.31) | 1.01(0.87,1.18) | 1.02(0.91,1.14) | 0.99(0.88,1.12) |
|  | 3^rd^ tertile | 1.38(1.18,1.61) | 1.07(0.91,1.26) | 1.09(0.98,1.21) | 0.98(0.86,1.11) |
|  | 2^nd^ tertile | 1.07(0.88,1.29) | 1.13(0.96,1.33) | 1.22(1.07,1.39) | 1.13(0.99,1.28) |
|  | 3^rd^ tertile | 1.13(0.92,1.38) | 1.23(1.01,1.49) | 1.46(1.27,1.68) | 1.20(1.03,1.39) |
| Ind+EMR+AS | 2^nd^ tertile | 1.12(0.94,1.32) | 1.04(0.89,1.21) | 1.06(0.95,1.18) | 1.02(0.90,1.15) |
|  | 3^rd^ tertile | 1.40(1.20,1.63) | 1.11(0.94,1.30) | 1.14(1.03,1.27) | 1.01(0.89,1.14) |
|  | 2^nd^ tertile | 0.95(0.82,1.10) | 0.93(0.79,1.11) | 0.94(0.85,1.04) | 0.90(0.79,1.03) |
|  | 3^rd^ tertile | 1.00(0.84,1.21) | 0.88(0.72,1.08) | 0.97(0.86,1.11) | 0.85(0.72,0.99) |
| Ind+CR+AS | 2^nd^ tertile | 1.13(0.94,1.37) | 1.13(0.96,1.33) | 1.25(1.09,1.42) | 1.11(0.98,1.26) |
|  | 3^rd^ tertile | 1.22(0.99,1.49) | 1.23(1.01,1.49) | 1.49(1.30,1.71) | 1.16(1.00,1.35) |
|  | 2^nd^ tertile | 0.96(0.83,1.12) | 0.95(0.80,1.13) | 0.96(0.87,1.07) | 0.92(0.80,1.04) |
|  | 3^rd^ tertile | 1.04(0.87,1.25) | 0.93(0.75,1.14) | 1.03(0.90,1.17) | 0.88(0.75,1.03) |
| Ind+EMR+CR+AS | 2^nd^ tertile | 1.11(0.94,1.31) | 1.02(0.87,1.19) | 1.02(0.91,1.14) | 1.00(0.89,1.13) |
|  | 3^rd^ tertile | 1.38(1.18,1.61) | 1.07(0.91,1.26) | 1.08(0.97,1.21) | 0.98(0.86,1.12) |
|  | 2^nd^ tertile | 1.08(0.89,1.31) | 1.12(0.95,1.32) | 1.23(1.08,1.41) | 1.11(0.98,1.27) |
|  | 3^rd^ tertile | 1.13(0.92,1.39) | 1.21(0.99,1.48) | 1.46(1.27,1.69) | 1.16(1.00,1.36) |
|  | 2^nd^ tertile | 0.96(0.82,1.11) | 0.96(0.80,1.13) | 0.96(0.87,1.07) | 0.92(0.80,1.04) |
|  | 3^rd^ tertile | 1.02(0.85,1.23) | 0.92(0.75,1.14) | 1.02(0.90,1.16) | 0.88(0.75,1.03) |

Table S7. Cox regressions for male preventable death (total), separately by young and old (aged 16-44 and 45-59 years respectively, in 2001). EMR denotes extrinsic mortality rate, CR-crime rate and AS- adult sex ratio. Models also control for age, marital/cohabiting status, dependent children in the household, community background and residence type.

|  |  | Preventable deaths | | | |
| --- | --- | --- | --- | --- | --- |
|  |  | Young  Pr3 | | Old  Pr3 | |
|  |  | Low SEP | High SEP | Low SEP | High SEP |
| Ind+EMR | 2^nd^ tertile | 0.97(0.78,1.21) | 1.01(0.81,1.25) | 1.06(0.91,1.23) | 1.11(0.94,1.31) |
|  | 3^rd^ tertile | 0.87(0.70,1.07) | 1.15(0.92,1.43) | 1.20(1.05,1.38) | 1.19(1.00,1.42) |
| Ind+CR | 2^nd^ tertile | 0.92(0.70,1.22) | 1.08(0.86,1.34) | 1.10(0.93,1.31) | 1.13(0.95,1.35) |
|  | 3^rd^ tertile | 1.10(0.82,1.46) | 0.86(0.65,1.12) | 1.20(1.00,1.43) | 1.25(1.02,1.54) |
| Ind+AS | 2^nd^ tertile | 0.98(0.80,1.21) | 0.92(0.73,1.16) | 0.94(0.82,1.07) | 0.97(0.81,1.16) |
|  | 3^rd^ tertile | 1.02(0.78,1.33) | 1.06(0.80,1.40) | 0.89(0.75,1.06) | 1.09(0.81,1.16) |
| Ind+EMR+CR | 2^nd^ tertile | 0.95(0.76,1.19) | 1.03(0.83,1.28) | 1.04(0.90,1.21) | 1.09(0.92,1.29) |
|  | 3^rd^ tertile | 0.85(0.69,1.05) | 1.18(0.94,1.47) | 1.18(1.03,1.36) | 1.16(0.97,1.38) |
|  | 2^nd^ tertile | 0.94(0.71,1.25) | 1.06(0.84,1.32) | 1.08(0.91,1.28) | 1.12(0.94,1.33) |
|  | 3^rd^ tertile | 1.14(0.85,1.52) | 0.83(0.63,1.09) | 1.15(0.96,1.38) | 1.22(0.99,1.50) |
| Ind+EMR+AS | 2^nd^ tertile | 0.97(0.78,1.21) | 1.00(0.80,1.24) | 1.07(0.92,1.24) | 1.10(0.93,1.30) |
|  | 3^rd^ tertile | 0.87(0.70,1.07) | 1.14(0.91,1.41) | 1.21(1.05,1.39) | 1.18(0.99,1.41) |
|  | 2^nd^ tertile | 0.98(0.80,1.21) | 0.93(0.74,1.17) | 0.94(0.83,1.08) | 0.98(0.81,1.17) |
|  | 3^rd^ tertile | 1.02(0.78,1.34) | 1.07(0.81,1.41) | 0.89(0.75,1.06) | 1.08(0.87,1.34) |
| Ind+CR+AS | 2^nd^ tertile | 0.93(0.70,1.23) | 1.09(0.87,1.36) | 1.09(0.92,1.29) | 1.16(0.97,1.38) |
|  | 3^rd^ tertile | 1.10(0.82,1.47) | 0.86(0.65,1.13) | 1.18(0.99,1.41) | 1.29(1.04,1.59) |
|  | 2^nd^ tertile | 1.00(0.81,1.23) | 0.89(0.71,1.13) | 0.95(0.83,1.09) | 1.00(0.83,1.20) |
|  | 3^rd^ tertile | 1.02(0.78,1.34) | 1.04(0.78,1.38) | 0.91(0.76,1.08) | 1.15(0.93,1.43) |
| Ind+EMR+CR+AS | 2^nd^ tertile | 0.95(0.76,1.19) | 1.02(0.82,1.28) | 1.05(0.91,1.23) | 1.08(0.91,1.28) |
|  | 3^rd^ tertile | 0.85(0.69,1.05) | 1.17(0.93,1.46) | 1.19(1.03,1.37) | 1.14(0.96,1.37) |
|  | 2^nd^ tertile | 0.95(0.71,1.26) | 1.07(0.85,1.34) | 1.07(0.90,1.27) | 1.14(0.95,1.36) |
|  | 3^rd^ tertile | 1.14(0.85,1.53) | 0.83(0.63,1.10) | 1.14(0.95,1.36) | 1.25(1.01,1.55) |
|  | 2^nd^ tertile | 1.00(0.81,1.24) | 0.90(0.71,1.14) | 0.95(0.83,1.09) | 1.00(0.83,1.20) |
|  | 3^rd^ tertile | 1.03(0.78,1.35) | 1.03(0.78,1.37) | 0.90(0.76,1.08) | 1.14(0.91,1.42) |

Table S8. Cox regressions for female preventable death (total), separately by young and old (aged 16-44 and 45-59 years respectively, in 2001). EMR denotes extrinsic mortality rate, CR-crime rate and AS- adult sex ratio. Models also control for age, marital/cohabiting status, dependent children in the household, community background and residence type.

| Table S9. Cox regressions with both sexes, for accidental/suicide deaths, alcohol related deaths, other preventable diseases, and all preventable deaths. HR- Hazard ratio, CI- confidence interval. AIC- Akaike Information Criterion | | | | | |
| --- | --- | --- | --- | --- | --- |
|  |  | Accident/suicide/  alcohol  Men 16-44 | Other  preventable  Men 16-44 | Accident/suicide/  alcohol  Women 16-44 | Other  preventable  Women 16-44 |
|  |  | **HR(95% CI)** | **HR(95% CI)** | **HR(95% CI)** | **HR(95% CI)** |
| **Age**  (16-19) | 20-24 | 0.78(0.62,0.97) | 1.04(0.68,1.59) | 0.68(0.41,1.12) | 1.49(0.83,2.67) |
|  | 25-29 | 0.75(0.59,0.96) | 1.26(0.81,1.98) | 0.98(0.59,1.62) | 2.13(1.16,3.91) |
|  | 30-34 | 0.91(0.71,1.15) | 1.49(0.96,2.32) | 1.38(0.84,2.27) | 3.56(1.97,6.43) |
|  | 35-39 | 1.11(0.88,1.41) | 2.88(1.88,4.41) | 1.61(0.98,2.65) | 4.68(2.60,8.43) |
|  | 40-44 | 1.24(0.97,1.58) | 5.09(3.33,7.77) | 2.03(1.23,3.34) | 9.62(5.38,17.21) |
| **Marital status**  (married/cohabiting) | Single | 1.20(0.98,1.46) | 1.31(0.99,1.73) | 1.08(0.81,1.45) | 1.03(0.77,1.38) |
|  | Sep/div/widowed | 1.57(1.26,1.95) | 1.17(0.86,1.59) | 1.97(1.53,2.54) | 1.03(0.77,1.38) |
| **Dependent children in household**  (1or >) | None (&age<=44) | 1.29(1.08,1.55) | 1.14(0.90,1.45) | 1.92(1.53,2.54) | 1.31(1.04,1.65) |
|  | None (&age>=45) | 1.21(0.96,1.53) | 1.07(0.78,1.48) | 1.76(1.22,2.54) | 2.36(1.68,3.32) |
|  | Cores. with parents | 1.43(0.98,2.09) | 1.47(0.98,2.23) | 1.81(1.05,3.12) | 0.93(0.53,1.64) |
| **Education**  (none) | University degree | 0.48(0.38,0.60) | 0.67(0.51,0.86) | 0.53(0.37,0.77) | 0.83(0.61,1.13) |
|  | 2+ A-levels | 0.80(0.65,1.00) | 0.99(0.74,1.32) | 0.56(0.37,0.84) | 0.88(0.62,1.24) |
|  | 1 A-level/GCSEs | 0.92(0.81,1.04) | 0.83(0.70,1.00) | 0.88(0.72,1.09) | 0.94(0.76,1.15) |
| **Economic activity** (active) | Unemployed | 1.60(1.34,1.91) | 1.24(0.91,1.69) | 1.84(1.24,2.75) | 1.51(0.98,2.35) |
|  | Student | 0.58(0.44,0.75) | 1.21(0.80,1.82) | 0.77(0.47,1.26) | 0.77(0.44,1.35) |
|  | Retired | 1.46(0.36,5.87) | 4.41(1.64,11.87) | DISCLOSIVE | 5.99(1.91,18.78) |
|  | Homemaker | 1.66(1.18,2.32) | 2.04(1.31,3.18) | 1.75(1.33,2.31) | 1.04(0.77,1.39) |
|  | Permanently ill | 3.00(2.55,3.52) | 4.55(3.70,5.59) | 3.99(3.08,5.18) | 4.56(3.62,5.75) |
|  | Other inactive | 2.47(2.02,3.02) | 2.08(1.49,2.91) | 1.78(1.23,2.55) | 2.08(1.48,2.92) |
| **Tenure**  (owner occupied) | Privately rented | 1.10(0.90,1.33) | 0.88(0.66,1.18) | 1.40(1.04,1.89) | 0.99(0.69,1.40) |
|  | Social housing | 1.34(1.16,1.55) | 0.99(0.80,1.22) | 1.28(0.99,1.64) | 1.39(1.09,1.77) |
| **Car access**  (0) | 1 | 0.59(0.51,0.68) | 0.82(0.66,1.02) | 0.66(0.52,0.84) | 0.86(0.67,1.10) |
|  | 2 or more | 0.46(0.38,0.54) | 0.69(0.53,0.89) | 0.56(0.41,0.77) | 0.80(0.59,1.04) |
| **Religion**  (Protestant) | Catholic | 1.01(0.90,1.12) | 0.89(0.76,1.04) | 1.02(0.85,1.22) | 0.87(0.73,1.04) |
|  | None/other | 0.74(0.51,1.06) | 1.31(0.89,1.94) | 1.55(0.94,2.54) | 0.61(0.29,1.29) |
| **Residence**  (Belfast) | Derry | 0.65(0.50,0.85) | 1.09(0.80,1.48) | 0.78(0.52,1.17) | 1.24(0.88,1.74) |
|  | Town | 1.09(0.95,1.24) | 0.91(0.75,1.09) | 1.19(0.96,1.48) | 1.08(0.88,1.34) |
|  | Rural areas | 1.19(1.04,1.36) | 0.87(0.72,1.04) | 1.02(0.81,1.30) | 0.98(0.78,1.22) |
| **AIC** |  | 35403.6 | 17966.8 | 12429.0 | 13616.6 |

|  |  | Main model | Interaction model |
| --- | --- | --- | --- |
|  |  | **HR(95% CI)** | **HR(95% CI)** |
| **Age**  (16-19) | 20-24 | 0.79(0.65,0.97) | 0.80(0.65,0.97) |
|  | 25-29 | 0.79(0.64,0.98) | 0.79(0.64,0.98) |
|  | 30-34 | 0.98(0.79,1.21) | 0.98(0.79,1.21) |
|  | 35-39 | 1.39(1.12,1.71) | 1.39(1.13,1.71) |
|  | 40-44 | 1.88(1.52,2.31) | 1.88(1.53,2.32) |
| **Marital status**  (married/cohabiting) | Single | 1.35(1.14,1.58) | 1.34(1.14,1.58) |
|  | Sep/div/widowed | 1.51(1.27,1.80) | 1.51(1.26,1.80) |
| **Dependent children in household**  (1or >) | None (&age<=44) | 1.24(1.08,1.44) | 1.24(1.08,1.44) |
|  | None (&age>=45) | 1.51(1.15,2.00) | 1.51(1.14,2.00) |
|  | Cores. with parents | 1.08(0.90,1.31) | 1.08(0.90,1.31) |
| **Economic activity**  (active) | Unemployed | 1.71(1.47,1.99) | 1.70(1.46,1.98) |
|  | Student | 0.71(0.57,0.89) | 0.71(0.57,0.89) |
|  | Retired | 2.80(1.26,6.27) | 2.79(1.25,6.24) |
|  | Homemaker | 2.00(1.53,2.65) | 2.00(1.53,2.62) |
|  | Permanently ill | 3.95(3.49,4.47) | 3.94(3.48,4.46) |
|  | Other inactive | 2.62(2.20,3.12) | 2.62(2.20,3.11) |
| **SEP** | Low SEP | 1.63(1.48,1.80) | 1.43(1.21,1.70) |
| **Religion**  (Protestant) | Catholic | 0.98(0.89,1.07) | 0.98(0.89,1.07) |
|  | None/other | 0.92(0.70,1.21) | 0.92(0.70,1.21) |
| **Residence**  (Belfast) | Derry | 0.83(0.68,1.02) | 0.84(0.69,1.03) |
|  | Town | 1.04(0.93,1.16) | 1.05(0.94,1.18) |
|  | Rural areas | 1.02(0.92,1.14) | 1.03(0.93,1.15) |
| **EMR** | 2^nd^ tertile | 1.07(0.95,1.19) | 1.03(0.89,1.20) |
| (1^st^ tertile) | 3^rd^ tertile | 1.27(1.14,1.42) | 1.10(0.94,1.29) |
| **EMR x SEP** | Low SEP x 2^nd^ tertile | - | 1.09(0.87,1.36) |
|  | Low SEP x 3^rd^ tertile | - | 1.31(1.05,1.62) |
| **AIC** |  | 51614.8 | 51612.3 |
| Table S10. Cox regressions for male preventable deaths (age 16-44), interaction between extrinsic mortality rate and socioeconomic position (EMR and SEP). *n*=317516, deaths=2095. SEP-socioeconomic position, EMR- Extrinsic mortality rate. HR- Hazard ratio, CI- confidence interval. AIC- Akaike Information Criterion. | | | |
